# Is the Autism-Spectrum Quotient a Valid Measure of Traits Associated with the Autism Spectrum? A Rasch Validation in Adults with and Without Autism Spectrum Disorders

**DOI:** 10.1007/s10803-017-3128-y

**Published:** 2017-04-19

**Authors:** Lars-Olov Lundqvist, Helen Lindner

**Affiliations:** 1 University Health Care Research Center, Faculty of Medicine and Health, Örebro University, P.O. Box 1613, 701 16 Örebro, Sweden; 2 School of Health Sciences, Örebro University, Örebro, Sweden

**Keywords:** Autistic traits, Adults, Autism-Spectrum Quotient, Rasch model

## Abstract

The Autism-Spectrum Quotient (AQ) is among the most widely used scales assessing autistic traits in the general population. However, some aspects of the AQ are questionable. To test its scale properties, the AQ was translated into Swedish, and data were collected from 349 adults, 130 with autism spectrum disorder (ASD) and 219 without ASD, and analysed with Rasch. Several scale properties of the AQ were satisfactory but it did not meet the criterion of a unidimensional measure of autistic traits. The Rasch analysis showed that the 50-item AQ could be reduced to a 12-item subset with little loss of explanatory power, with the potential to efficiently measure the degree to which adults with and without ASD show autistic traits.

## Introduction

Autism spectrum disorder (ASD) is characterized by persisting deficits in social communication and interaction, alongside repetitive, stereotyped behavior and restricted interests (APA, 2013). The number of adults diagnosed with ASD has increased dramatically in the past decade and ASD now accounts for a large burden on health care (Fombonne 2009; Keyes et al. 2012). The global prevalence varies greatly but is approximately 1% (Elsabbagh et al. 2012), with 1.8% in men and 0.2% in women (Brugha et al. 2011). Autistic traits have moderate to high heritability, are highly stable, and distributed on a continuum in the general population, where ASD is at one extreme of the population distribution (Hoekstra et al. 2007; Robinson et al. 2011).

Studying autistic traits can give further insight into how they relate to mental processes (Kuo et al. 2014), individual differences (Rivet and Matson 2011), and psychiatric disorders such as anxiety and depression (Rosbrook and Whittinham 2010). Screening for autistic traits in the general population may be helpful in epidemiological research because it may provide necessary sample size to investigate relationships between autism phenotype severity and theoretically important factors. Furthermore, examining autistic traits in general population samples can serve as ‘analogue studies’ for ASD, providing access to larger, more easily accessible samples and thus allowing more complex statistical analyses to be conducted (e.g. Jackson and Dritschel 2016; Kunihira et al. 2006).

Among the variety of screening tools developed to quantify autistic traits, over the past decade the most commonly used is probably the Autism-Spectrum Quotient (AQ; Baron-Cohen et al. 2001a). The AQ has been used to screen clinical samples (Woodbury-Smith et al. 2005) and to predict performance on cognitive tasks (Stewart et al. 2009), social cognition (Baron-Cohen et al. 2001b), spontaneous facial mimicry (Hermans et al. 2009), gaze preference to social and non-social stimuli (Bayliss and Tipper 2005), and auditory speech perception (Stewart and Ota 2008).

The AQ is a self-administered questionnaire for measuring the degree to which adults with normal intelligence show autistic traits. It consists of 50 questions, with 10 questions assessing five different domains relevant for autistic traits (*social skill, attention switching, attention to detail, communication, and imagination*). Adequate test–retest reliability has been shown in the AQ (Baron-Cohen et al. 2001a) and the AQ sum scores are normally distributed in the general population (Hurst et al. 2007). Cross-cultural equivalence in Dutch and Japanese samples has also been shown (Hoekstra et al. 2008; Kurita et al. 2005; Wakabayashi et al. 2006).

However, some aspects of the AQ are still questionable. Baron-Cohen et al. (2001a) originally proposed a unidimensional structure of the AQ based on descriptive item analysis and sum score distribution across ASD and non-ASD groups. The sum score is by far the most commonly used AQ result, yet Baron-Cohen et al. (2001a) only found adequate internal consistency (defined as Cronbach’s alpha above 0.70; Nunnally and Bernstein 1994) in one of the five autism trait domains in the AQ. Low Cronbach’s alpha indicates a lack of correlation between the items in a scale, which suggests deviation from unidimensionality. The low degree of internal consistency in the AQ has been extensively replicated (e.g., Austin 2005; Hoekstra et al. 2008; Hurst et al. 2007; Kloosterman et al. 2011; Stewart and Austin 2009).

To date, studies using more advanced statistical methods, such as factor analysis, have demonstrated that the AQ may consists of five (Kloosterman et al. 2011; Lau et al. 2013), four (Stewart and Austin 2009), three (Austin 2005; Hurst et al. 2007) or two (Hoekstra et al. 2008) dimensions. The two-factor model (actually two higher-order factors and four primary factors) was confirmed in a validation of a 28-item short form of the AQ (Hoekstra et al. 2011). Thus, the unidimensional structure assumed by Baron-Cohen et al. (2001a) has not been replicated.

A common feature of previous studies is that the psychometric analyses are mostly based on non-ASD samples. The choice of mainly student samples may be reasonable, given that the AQ is directed towards autistic traits in the general population. However, the feasibility of the AQ and the theoretical basis of an autistic trait continuum require that the properties of the AQ are similar among those with and without ASD.

Another common feature of these studies is that they apply classical test theory techniques, such as principal component analysis, exploratory factor analysis or confirmatory factor analysis. As shown by Gorsuch (1997), factor analysis on ordinal data, if treated as interval data, can result in spurious factors. In addition, item distributions may differ from each other and therefore items will tend to load on the same factor as other items with similar distributions. One will thus make erroneous conclusions about the scale, especially when sum score, as in AQ, is used to define the degree of an underlying trait. Consistent with this, Stewart and Austin (2009) noted that their initial exploratory factor analysis suggested a large number of poorly defined factors. Consequently, these numerous factors may possibly reflect distribution properties and not the underlying construct being measured. Therefore, we will take a different approach in the present study and examine the dimensionality of AQ using Rasch analysis.

Rasch models (Rasch 1960) have currently been applied in the development and validation of unidimensional scales with interval scale properties based on frequency questions or Likert items. They facilitate calibration of the observed test values with the underlying latent property (Linacre 1994). Rasch analysis can thus determine the degree to which items in the AQ accurately characterize autistic traits. Rasch models facilitate analysis of whether an instrument meets the requirements of invariance; for instance whether the scale works in a similar manner among men and women with and without ASD. Finally, the Rasch model is a method to validate the interval properties of a scale. An advantage of Rasch analysis is that it makes no assumptions about the distribution of the latent property, whereas in classical test theory techniques, normally distributed latent variables are required. Hence, the aim of the study was to test the scale properties of the Swedish AQ using Rasch analysis.

## Methods

### Participants

Two samples, an ASD group and a non-ASD group, were recruited for this study. The ASD group was recruited from the Centre for Adult Habilitation, Region Örebro County, Sweden. A total of 401 adults diagnosed with ASD and without intellectual impairment (i.e., IQ > 70) were invited to participate and 130 of them volunteered (68 men and 62 women, age 18–62, mean = 29.3 years, SD = 9.9). No age difference was found between the participants and the non-participants; however, the proportion of participating men (28%) was significantly lower than the proportion of participating women (40%) (χ^2^ = 6.25, p < 0.05).

The non-ASD group consisted of 219 university students recruited from various departments at Örebro University (93 men and 126 women, age 18–55 years, mean = 23.8 years, SD = 5.7). None of them reported having an ASD diagnosis. No age difference was found between men and women (t_(217)_ = 0.68, p = 0.50) and the sex ratio of the sample was equivalent to that of the university (i.e., 60% women).

The ASD and non-ASD groups differed in regard to sex and age. The ASD group had significantly more men than the non-ASD group (χ^2^ = 9.43, p < 0.01) and the ASD group was on average older than the non-ASD group (t_(347)_ = 9.06, p < 0.001).

### The Autism-Spectrum Quotient

The Autism-Spectrum Quotient (AQ; Baron-Cohen et al. 2001a) is a 50-item self-report questionnaire for measuring the degree to which an adult with normal intelligence has the traits associated with the autistic spectrum. The items, which are given in Table 3, assess five different domains (10 items per domain): *social skill, attention switching, attention to detail, communication*, and *imagination*. All items are scored on a four-point rating scale ranging from 1 = *definitely agree* to 4 = *definitely disagree*. The scorings are reversed (from 4 = *definitely agree* to 1 = *definitely disagree*) for the items in which an “agree” response indicates an autistic trait. The following items were reversed: 2, 4, 5, 6, 7, 9, 12, 13, 16, 18, 19, 20, 21, 22, 23, 26, 33, 35, 39, 41, 42, 43, 45, and 46. All item scores are summed; thus, AQ sum score can vary between 50 (at the lowest extreme of the autistic trait continuum) and 200 (at the highest extreme of the autistic trait continuum).

The AQ was translated into Swedish after permission from Professor Simon Baron-Cohen. The translation was performed independently by two professional translators. The two translations were compared and the few minor discrepancies that emerged, which consisted of different choices of synonymous words or sentence structure, were discussed with the translators. Subsequently, a third professional translator translated the Swedish version back into English to confirm equivalence with the original. Hence, the Swedish version of AQ is linguistically similar to the English original. The Swedish translation is available from the first author.

### Procedure

The adults with ASD received the study information, the study consent form, the AQ questionnaire, and a prepaid envelope by post. The students (non-ASD group) were informed verbally about the study and completed the AQ questionnaire during lectures. No course credit was received.

### Data Analysis

IBM SPSS Statistics version 22 (IBM Corp, Armonk, NY) was used to summarize participant characteristics and to evaluate group differences using t-tests. A *p* value below 0.05 was regarded as significant. The AQ rank-ordered scores were analyzed using Rasch rating scale model with Winsteps 3.81.0 (Linacre 2014). Detailed explanation of Rasch models is given elsewhere (Engelhard 2013). In brief, Rasch analysis converts rank-ordered data into interval logit measures, giving each person and each item a logit measure. Logit stands for Log-Odds Unit and form an equal interval linear scale. The logit scale is unaffected by variations in the distribution of measures and independent of the particular items included in a test or the particular samplings of people (Wright 1993). Thus, an ‘AQ person measure’ represents the degree to which a person shows autistic traits (the higher the logits, the higher the degree of autistic traits). An ‘AQ item measure’ represents how difficult any particular item may be to endorse given a specific degree of autistic traits (the higher the logits, the more difficult to endorse). Rasch analysis enables the researchers to identify whether any items are misleading and whether the rating categories have been used as intended by the instrument developer.

#### Rating Categories

The four rating categories were examined according to four criteria (Linacre 2002): *(i)* there should be at least 10 responses in each rating category, *(ii)* the average AQ person measure should be lower in a category representing low AQ than in one representing high AQ, *(iii)* the transition point between each two categories (threshold) should follow an increasing level of the underlying autistic trait, and *(iv)* the category outfit mean square should be less than 2.0. The rating scale graphs generated by Winsteps were used to examine the ordering of thresholds and how the rating categories were positioned along the latent variable.

#### Item Properties

Point–measure correlations, local item independence, and fit statistics were used to examine the item properties. Point–measure correlation of each item reports the relationship between the group’s performance on the item and the group’s performance on the whole instrument. All items are expected to correlate positively in the direction of the latent variable, if any items show negative correlations it is assumed that these items are considered invalid. Local item independence assessed whether responses to any item were unrelated to any other item when trait level was controlled; thus, the endorsement of any item should not affect the probability of endorsement of the other items. Violation of local item independence may affect parameter estimates. An item residual correlation of at least 0.7 (i.e., common variance approximately 0.50) was set as a criterion for item dependency (Linacre 2009). Fit statistics detect the extent to which the response pattern observed in the data matches the one expected by the model. In this study, an item was considered as misfit if infit and outfit mean square was greater than 1.50.

#### Differential Item Functioning

Differential item functioning (DIF) was used to examine whether an item performed differently for the ASD group than for the non-ASD group. For this study, item DIF was considered present if the difference between two groups on an item measure was 0.5 logits or more and reached significance (p < 0.05) in a t test (Karami 2012).

#### Scale Reliability

Scale reliability was evaluated in terms of person reliability, an index similar to Cronbach’s alpha: for the range 0–1, coefficients above 0.70 are considered as a minimum for group use and coefficients above 0.85 for individual use (Tennant and Conaghan 2007).

#### Unidimensionality

Principal components analysis of residuals was used to examine whether the five AQ domains measure different dimensions or work together to measure one dimension. We used two criteria: at least 50% of the total variance should be explained by the first latent variable and any additional factor should explain less than 5% of the remaining variance after removal of the first latent variable (Linacre 2009).

#### Targeting

We explored the potential use of the AQ to measure a clinical population by examining the targeting of item difficulty (not too easy, not too hard) to the individual’s trait level in the person–item map. The map orders person and item measures along the same scale, which enables us to examine whether the AQ has enough items to discriminate people with different levels of autistic traits. The item difficulty range is expected to match the range of autistic trait levels in the ASD group. A value around zero thus indicates that the items are well targeted for the people in the sample (Tennant and Conaghan 2007).

#### Sensitivity and Specificity

Sensitivity and specificity of the AQ as a screening tool for ASD was evaluated using the receiver operating characteristic (ROC) curve and area under the curve (AUC) calculated for the full AQ scale and the five AQ domains. The Youden index (Youden 1950), which is the point at which the tangent to the ROC curve is parallel to the chance line, was used to find the optimal cut-off scores. This index has been used in the development of diagnostic assessments for ASD (Cohen et al. 2010) and is regarded as one of the most stringent statistical method to identify a cut-off or threshold in diagnostic measures.

## Results

### Person Measures

Mean AQ person measure for the ASD group was significantly higher (t_(347)_ = 15.02, p < 0.01) than the mean AQ person measure for the non-ASD group (Table 1). No significant differences between men and women were found in either group.

**Table 1:** Mean AQ sum scores and mean person measures

	ASD		Non-ASD	
	Logits	Sum score	Logits	Sum score
All				
Mean	0.07	127.73	−0.53	102.83
SD	0.46	18.81	0.29	11.18
Men				
Mean	0.03	126.22	−0.49	104.33
SD	0.42	17.37	0.28	11.27
Women				
Mean	0.10	129.37	−0.56	101.72
SD	0.50	20.28	0.29	11.03

*Logits* log-odd units, *AQ* Autism-Spectrum Quotient, *ASD* autism spectrum disorder

### Rating Categories

**Fig. 1:**
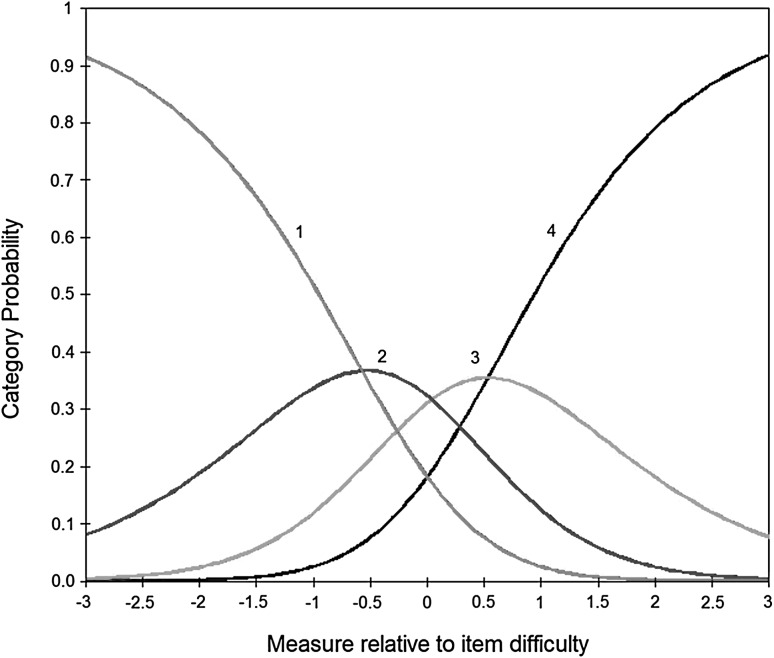
The category probability *curves* of the AQ, illustrating the range over which each of the four categories is most likely to be chosen. Boundaries occur at points along the scale where the category most likely to be chosen changes from one to the next. The *1, 2, 3* and *4* category *curves* on the graph represent the four rating categories, from 1 = *definitely agree* to 4 = *definitely disagree*. The *x-axis* is AQ person measure minus AQ item measure in logits. (*AQ* Autism-Spectrum Quotient, *logit* log-odd unit)

Both groups fulfilled the rating scale criteria (Table 2). That is, there were more than 10 responses in each rating category, average person AQ measure increased with the rating category, thresholds were ordered, and category outfit mean square was below 2.0. The probability that an individual with a given autistic trait level will select a response category is shown in Fig. 1. For any given point along the x-axis (representing autistic trait continuum), the category most likely to be chosen by an individual is shown by the category curve with the highest probability. An optimally functioning scale should have each category most likely to be selected for an equal interval on the scale, which the AQ demonstrated.

**Table 2:** Summary statistics for the four AQ rating scale categories

	Category	Frequency of use (%)		Average AQ person measure (logits)		Threshold measure		Category infit mean square		Category outfit mean square	
		ASD	Non-ASD	ASD	Non-ASD	ASD	Non-ASD	ASD	Non-ASD	ASD	Non-ASD
1	Definitely agree	1507 (23)	3750 (34)	−1.77	−2.17	None	None	1.01	1.01	1.04	1.02
2	Slightly agree	1539 (24)	3850 (35)	−0.49	−0.59	−0.25	−0.84	0.93	0.94	0.90	0.90
3	Slightly disagree	1790 (28)	2333 (21)	0.47	0.61	−0.11	0.08	0.89	0.98	0.86	0.97
4	Definitely disagree	1662 (26)	1017 (9)	1.80	2.14	0.36	0.76	1.05	1.05	1.08	1.09

Frequency of use’: the number of persons rated in that category. ‘Average AQ person measure’: observed mean person measure (in logits) in each rating category. ‘Threshold measure’: the difficulty measure between every two adjacent categories. Infit mean square and outfit mean square examine the consistency of use of each rating category; this should not exceed 2.0. (AQ = Autism-Spectrum Quotient, ASD = autism spectrum disorder.)

### Item Properties

#### Point–Measure Correlations

Three items, 29 “I am not very good at remembering phone numbers”, 30 “I don’t usually notice small changes in a situation, or a person’s appearance”, and 49 “I am not very good at remembering people’s date of birth”, had point–measure correlations lower than zero (−0.02, −0.18, and −0.01, respectively). The negative correlations suggest that people with high scores on these items had a lower autistic trait level, not higher as was expected.

#### Local Item Independence

All items showed standardized residual correlations below 0.7. The greatest standardized residual correlations were between items 17 and 38 (0.58) and between items 44 and 47 (0.58).

#### Fit Statistics

As shown in Table 3, the logit measures of the 50 items ranged from 0.99, most difficult to endorse (item 09 “I am fascinated by dates”), to −1.11, easiest to endorse (item 30 “I don’t usually notice small changes in a situation, or a person’s appearance”), both in the domain *Attention to detail*. Five items were misfit: item 21 in *Imagination* and items 9, 29, 30, and 49 in *Attention to detail*.

**Table 3:** Item raw scores, logit measures and fit statistics for the Autism-Spectrum Quotient

	All			ASD		Non-ASD	
	Item logit measure	Infit mean square	Outfit mean square	Mean raw score	Item logit measure	Mean raw score	Item logit measure
Social skill							
1 I prefer to do things with others rather than on my own	−0.06	0.68	0.68	2.61	−0.06	2.06	−0.06
11 I find social situations easy	0.37	0.71	0.69	2.58	−0.03	1.53	0.73
13* I would rather go to a library than a party	0.53	1.25	1.16	2.55	0.01	1.37	1.10
15 I find myself drawn more strongly to people than to things	−0.03	0.71	0.71	2.80	−0.28	1.91	0.13
22* I find it hard to make new friends	0.47	1.02	0.98	2.55	0.00	1.43	0.93
36 I find it easy to work out what someone is thinking or feeling	−0.29	0.75	0.74	2.90	−0.39	2.21	−0.23
44 I enjoy social occasions	0.80	0.73	0.69	2.16	0.44	1.32	1.22
45* I find it difficult to work out people’s intentions	0.18	0.57	0.57	2.54	0.02	1.79	0.29
47 I enjoy meeting new people	0.45	0.71	0.69	2.48	0.08	1.49	0.80
48 I am a good diplomat	−0.08	0.91	0.93	2.48	0.08	2.17	−0.18
Attention switching							
2* I prefer to do things the same way over and over again	−0.24	0.62	0.62	2.60	−0.05	2.33	−0.35
4* I frequently get strongly absorbed in one thing	−0.45	1.01	1.02	2.93	−0.43	2.43	−0.45
10 I can easily keep track of several different people’s conversations	−0.28	0.81	0.81	2.99	-0.51	2.15	−0.15
16* I tend to have very strong interests	−0.47	0.90	0.92	2.82	−0.30	2.53	−0.56
25 It does not upset me if my daily routine is disturbed	−0.36	1.16	1.21	2.71	−0.17	2.44	−0.47
32 I find it easy to do more than one thing at once	−0.27	0.85	0.88	3.04	−0.57	2.11	−0.11
34 I enjoy doing things spontaneously	0.03	0.75	0.74	2.64	−0.09	1.93	0.11
37 If there is an interruption, I can switch back very quickly	−0.31	0.67	0.67	2.86	−0.35	2.26	−0.28
43* I like to plan any activities I participate in carefully	−0.43	0.84	0.83	2.96	−0.47	2.38	−0.43
46* New situations make me anxious	−0.42	0.76	0.76	3.08	−0.62	2.29	−0.31
Communication							
7* Other people frequently tell me that what I have said is impolite	0.85	1.04	1.02	1.91	0.76	1.43	0.93
17 I enjoy social chit-chat	−0.14	0.85	0.85	2.79	−0.27	2.07	− 0.06
18* When I talk, it isn’t always easy for others to get a word in edgeways	0.57	1.21	1.27	1.87	0.82	1.73	0.38
26* I don’t know how to keep a conversation going	0.17	0.87	0.86	2.59	−0.04	1.77	0.32
27 I find it easy to ‘‘read between the lines’’	−0.23	0.80	0.81	2.76	−0.23	2.21	−0.23
31 I know how to tell if someone listening to me is getting bored	0.06	0.81	0.81	2.67	−0.13	1.86	0.19
33* When I talk on the phone, I am not sure when it’s my turn to speak	0.85	1.05	0.99	1.93	0.73	1.42	0.96
35* I am often the last to understand the point of a joke	0.53	1.31	1.30	2.03	0.60	1.67	0.47
38 I am good at social chit-chat	−0.19	0.73	0.72	2.88	−0.38	2.08	−0.08
39* People tell me that I keep going on and on about the same thing	0.70	1.21	1.19	2.02	0.62	1.50	0.78
Imagination							
3 Trying to imagine something, I find it easy to create a picture in my mind	0.36	0.83	0.81	2.18	0.42	1.78	0.31
8 Reading a story, I can easily imagine what the characters might look like	0.31	1.04	1.02	2.25	0.35	1.79	0.29
14 I find making up stories easy	−0.52	1.34	1.39	2.55	0.00	2.75	−0.81
20* Reading a story, I find it difficult to work out the characters’ intentions	0.62	0.79	0.79	1.92	0.74	1.65	0.51
21* **I don’t particularly enjoy reading fiction**	0.13	1.65	1.65	2.30	0.29	2.00	0.02
24 I would rather go to the theatre than a museum	−0.52	1.25	1.25	3.15	−0.71	2.40	−0.43
40 When younger, I enjoyed playing games involving pretending with other children	−0.39	1.25	1.26	2.78	−0.26	2.43	−0.47
41* I like to collect information about categories of things	0.58	1.29	1.22	2.24	0.36	1.50	0.78
42* I find it difficult to imagine what it would be like to be someone else	0.08	1.05	1.06	2.38	0.19	2.01	0.01
50 I find it easy to play games with children that involve pretending	−0.59	1.03	1.02	2.99	−0.51	2.59	−0.63
Attention to detail							
5* I often notice small sounds when others do not	−0.26	1.15	1.15	2.89	−0.39	2.18	−0.19
6* I usually notice car number plates or similar strings of information	−0.30	1.25	1.27	2.74	−0.21	2.33	−0.42
9* **I am fascinated by dates**	0.99	1.53	1.58	1.58	1.27	1.51	0.84
12* I tend to notice details that others do not	−0.76	1.05	1.03	3.05	−0.58	2.79	−0.97
19* I am fascinated by numbers	0.40	1.39	1.39	1.77	0.69	1.97	0.04
23* I notice patterns in things all the time	−0.09	1.12	1.13	2.30	0.29	2.29	−0.37
28 I usually concentrate more on the whole picture, rather than the small details	−0.40	0.84	0.87	2.89	−0.40	2.37	−0.47
**29 I am not very good at remembering phone numbers**	−0.46	1.54	1.62	2.48	0.09	2.72	−0.88
**30 I don’t usually notice small changes in a situation, or a person’s appearance**	−1.11	1.44	1.92	3.02	−0.55	3.24	−1.59
**49 I am not very good at remembering people’s date of birth**	−0.41	1.58	1.71	2.48	0.08	2.63	−0.67

*Designates a reverse-scored item. Bold designates misfit items. Higher item logits denotes a more “difficult” item. Higher mean raw score denotes higher degree of autistic traits. (*Logit* log-odd unit, *ASD* autism spectrum disorder)

### Item DIF

Five items showed DIF between the ASD and non-ASD groups: items 13 (−1.09 logits), 22 (−0.93 logits), and 44 (0.79 logits) in the domain *Social skill*, item 14 (0.81 logits) in *Imagination* and item 19 (−0.91 logits) in *Attention to detail*. Note that items 13, 19, and 22 have reversed scoring. Given identical levels of autistic traits, items 13 “I would rather go to a library than a party”, 19 “I am fascinated by numbers”, and 22 “I find it hard to make new friends” were thus more likely to be endorsed by those in the ASD than those in the non-ASD group, whereas items 14 “I find making up stories easy” and 44 “I enjoy social occasions” were more likely to be endorsed by those in the non-ASD group.

### Unidimensionality

The principal components analysis of all 50 items showed that the AQ instrument did not fulfill the unidimensionality criteria. The raw variance explained by the measures (which should be above 50%) was 26.2% and the unexplained variance in first contrast (which should be below 5%) was 7.7%. Three clusters were formed and we repeated the analyses with each cluster of items. Only one cluster fulfilled both criteria, showing a raw variance explained by the measures of 52.8% and an unexplained variance in first contrast of 2.7%. This cluster consisted of 12 items: 11, 13, 22, 44, and 47 in the domain *Social skill*, 10, 32, 34, and 46 in *Attention switching*, and 17, 26, and 38 in *Communication*.

### Targeting

**Fig. 2:**
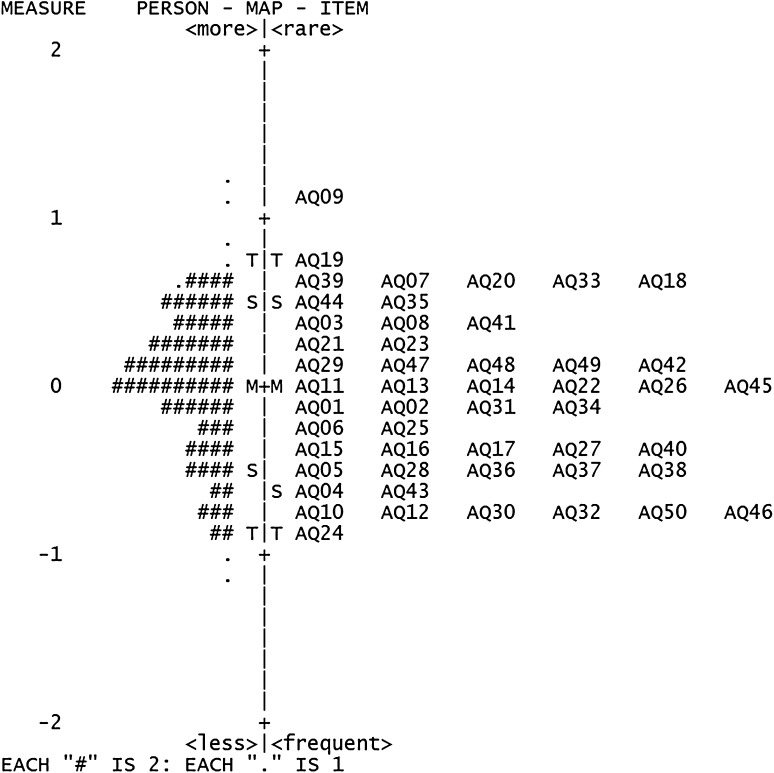
Person–item map for the ASD group: AQ person measures in relation to AQ item measures in logits. (*M* mean, *S* 1 standard deviation (SD) from the mean, *T* 2 SD from the mean, *AQ* Autism-Spectrum Quotient, *ASD* autism spectrum disorder)

**Fig. 3:**
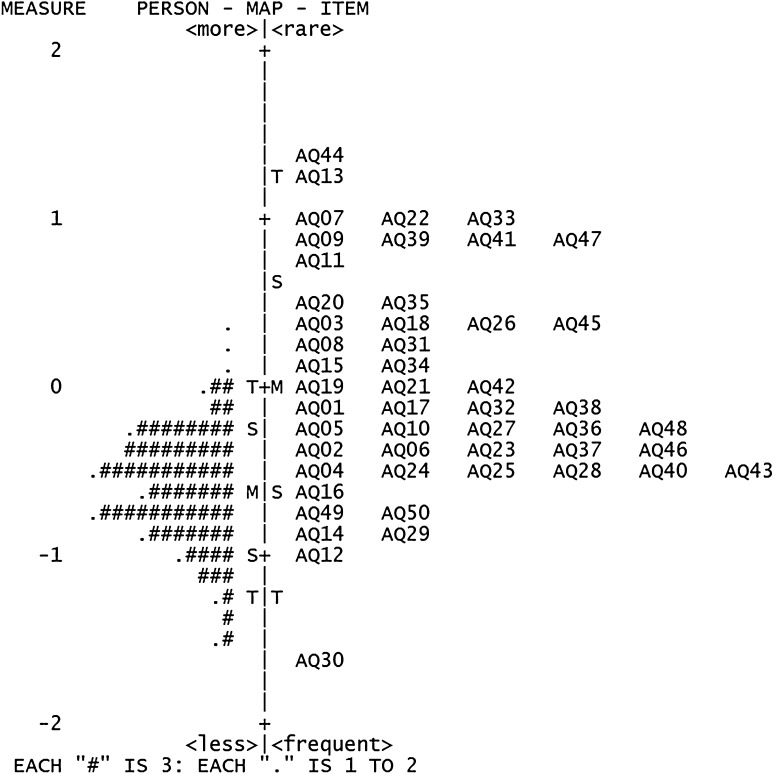
Person-item map for the non-ASD group: AQ person measures in relation to AQ item measures in logits. (*M* mean, *S* 1 standard deviation (SD) from the mean, *T* 2 SD from the mean. (*AQ* Autism-Spectrum Quotient, *ASD* autism spectrum disorder)

The range of AQ items targeted well, showing person means and item means close to each other, with a mean measure of −0.31. The targeting in the ASD group was excellent, with a mean measure of 0.04 (Fig. 2) whereas it was acceptable in the non-ASD group, as indicated by a mean measure of −0.61 (Fig. 3).

### Scale Reliability

Person separation was 2.52 and person reliability was 0.86. Item separation was 7.29 and item reliability was 0.98. There was a strong correlation between AQ logits and AQ sum score (r = 0.998, p < 0.001).

### Sensitivity and Specificity

**Fig. 4:**
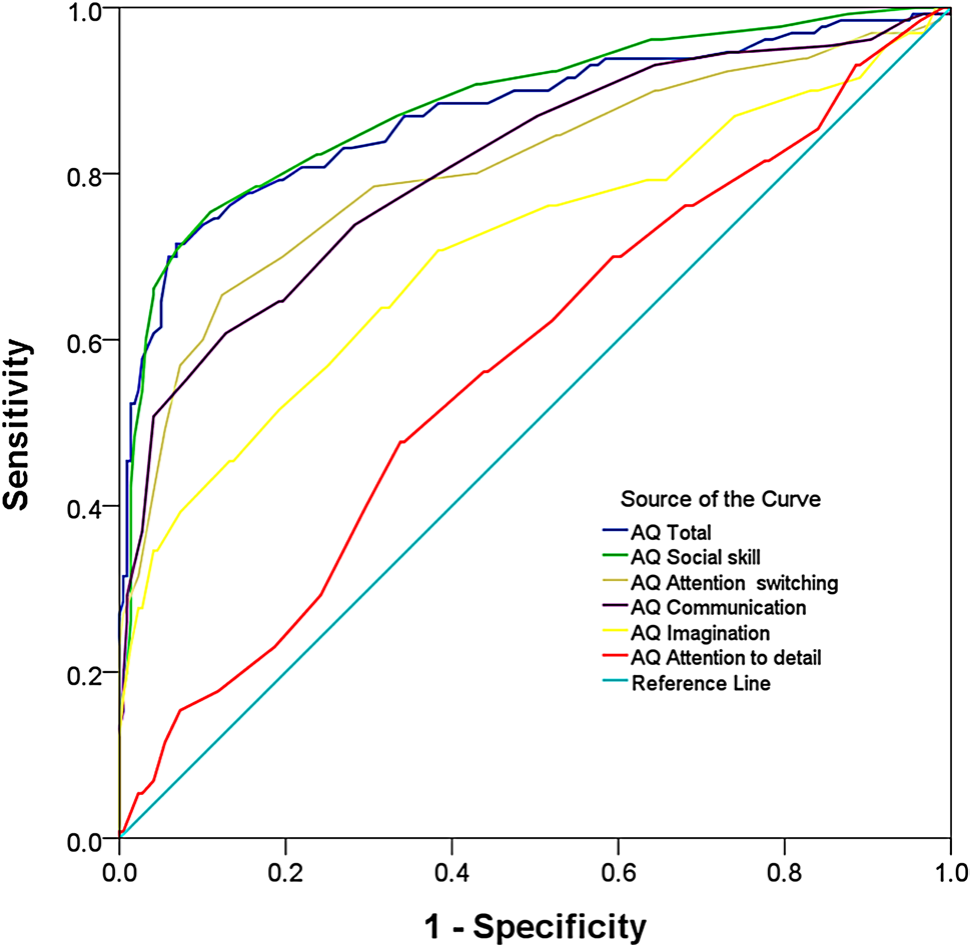
Receiver operating characteristic (ROC) curves illustrating the ability of the full AQ scale and AQ domains to identify any ASD cases at alternative cut-off points. Note that a perfect measure would have an area under the curve of 1.0, whereas a measure with no diagnostic value would have an area of 0.5, with the ROC curve laying on the diagonal. (*AQ* Autism-Spectrum Quotient, *ASD* autism spectrum disorder)

**Fig. 5:**
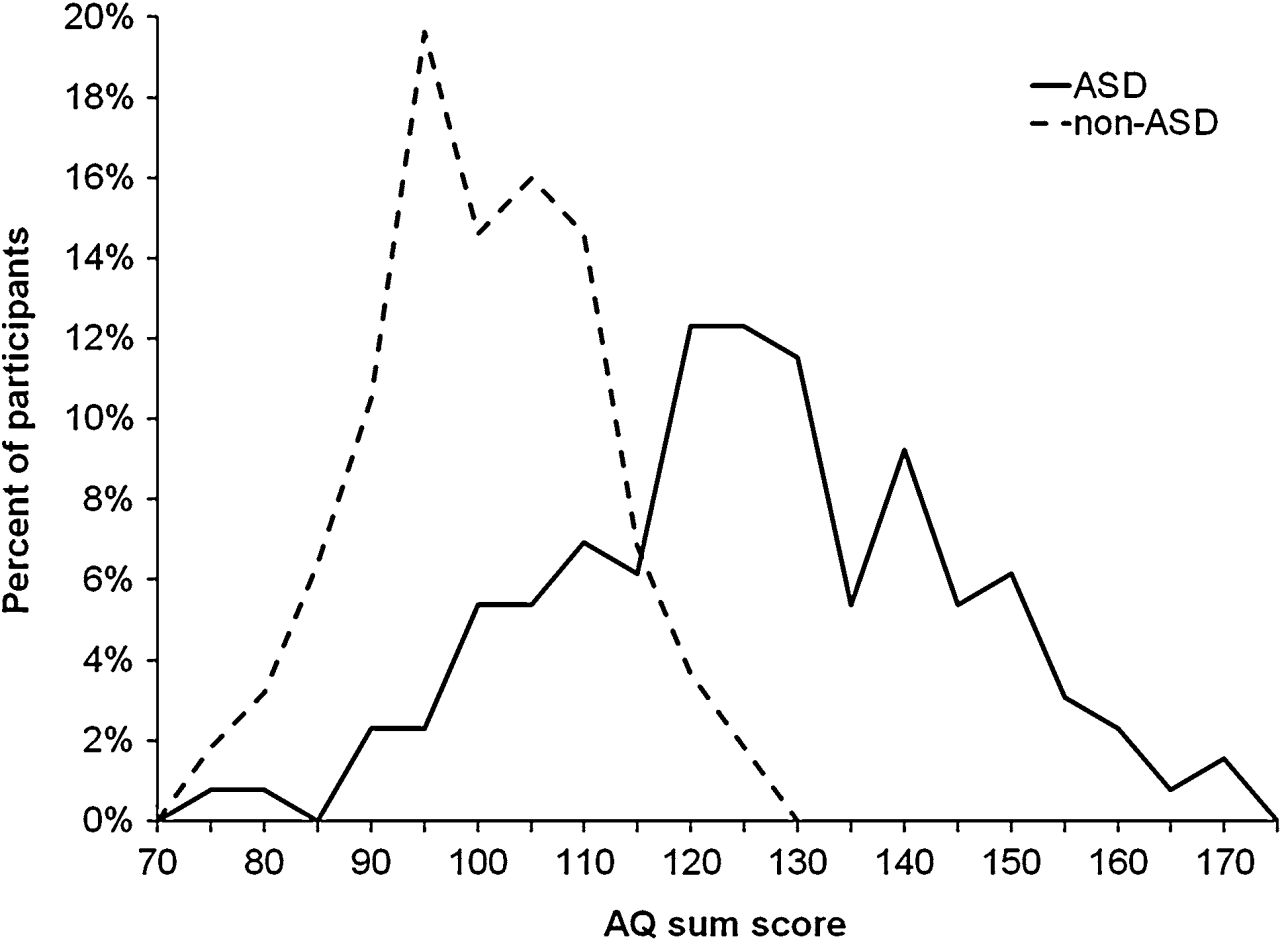
AQ sum scores in ASD and non-ASD participants (*AQ* Autism-Spectrum Quotient, *ASD* autism spectrum disorder)

The ROC curves for the full AQ scale and the five AQ domains are shown in Fig. 4. The AUC was significant for all domains, the sensitivity varied between 48 and 75%, and specificity varied between 66 and 93% (Table 4). A sum raw score of 118 was identified as the optimal screening cut-off score for the full AQ scale, where Youden’s index was 0.65 (95% CI 0.55–0.71). The correct classification was 85.2% (95% CI 80.6–88.1%), the positive predictive value was 0.85 (95% CI 0.79–0.91), and the negative predictive value was 0.85 (95% CI 0.82–0.87). The AQ sum score distribution for ASD and non-ASD participants is shown in Fig. 5.

**Table 4:** Area under the curve **(**AUC), sensitivity, and specificity for the Youden index based cut-off scores for the full AQ scale and five AQ domains

AQ	AUC^#^ (95% CI)	*p*	Sensitivity (95% CI)	Specificity (95% CI)	Cut-off
AQ total	0.87 (0.83–0.92)	<0.001	0.79 (0.71–0.86)	0.81 (0.75–0.86)	113
Social skills	0.88 (0.84–0.92)	<0.001	0.78 (0.70–0.85)	0.84 (0.78–0.88)	21
Attention switching	0.81 (0.75–0.86)	<0.001	0.70 (0.61–0.78)	0.80 (0.74–0.85)	26
Communication	0.81 (0.76–0.86)	<0.001	0.64 (0.56–0.73)	0.81 (0.75–0.86)	21
Imagination	0.71 (0.65–0.77)	<0.001	0.52 (0.43–0.60)	0.81 (0.75–0.86)	25
Attention to detail	0.57 (0.51–0.63)	0.028	0.48 (0.39–0.57)	0.66 (0.60–0.72)	26

*AQ* Autism-Spectrum Quotient

N = 349

^#^Null hypothesis true area = 0.5

## Discussion

The study tested the scale properties of the Swedish AQ using the Rasch rating scale model, with mixed results: several scale properties were good to excellent whereas others were poor. On the one hand, the AQ fulfilled the rating scale criteria, had minimal DIF, adequate item properties, adequate item and person separation and reliability, and excellent targeting for the ASD group; on the other hand, the AQ did not meet the criteria for a unidimensional scale.

In regard to item properties, five items were misfit and thus did not fit the expected model: item 21 in the domain *Imagination* and items 9, 29, 30, and 49 in *Attention to detail*. Three of the items (29, 30, and 49) had negative point–measure correlations, with the scoring orientation on these items opposite to the orientation of the latent variable (the degree of autistic traits). Reasons for negative point–measure correlations can, for instance, be person-specific knowledge, guessing, or reverse scoring. It is notable that all three items are negatively worded and that these items were also scored higher by the non-ASD group than the ASD group, suggesting that the items do not represent a measure of autistic traits and need revision. This is in line with previous studies finding low or negative domain loadings for these items (Austin 2005; Hoekstra et al. 2008; Hurst et al. 2007; Stewart and Austin 2009). It should be noted that in the development of the AQ, Baron–Cohen and colleagues (Baron–Cohen et al. 2001a) found that items 29 and 30 were scored higher by controls than adults with Asperger’s syndrome or high-functioning autism, but nevertheless were retained in order to reduce the group differences.

No item pair was locally dependent, although item residuals were moderately correlated between “*I enjoy social chit-chat*” (item 17) and “*I am good at social chit-chat*” (item 38), and between “*I enjoy social occasions*” (item 44) and “*I enjoy meeting new people*” (item 47). In both pairs, the items are similar in meaning. Even if they fit the model, use of highly similar worded items will boost the items’ correlation with the total score while providing no unique information about the responder. In the presence of local dependency, it is recommended that one of the similar items should be excluded due to potential redundancy.

Five of the 50 items showed DIF, three from the *Social skill* domain, one from the *Imagination* domain, and one from the *Attention to detail* domain. Interestingly, the DIF indicated that these items exaggerated the group differences in the expected direction. That is, people with ASD are expected to be less socially skilled and imaginative and more attentive to details than those without ASD; these items thus highlight the group differences more distinctly than the other items in the AQ. Absence of DIF is crucial for an adequate scale (Tennant and Conaghan 2007), but given this overestimation bias—that only five out of 50 items showed DIF and that all but one of these items were below 1 logit—it would appear that the AQ items, for all practical purposes, are adequate for people with as well as without ASD.

The AQ items targeted well at the individuals with ASD. However, as shown in the person–item maps, most of the non-ASD respondents were clustered at the lower end of the measures, indicating a low position on the autistic continuum, while many of the items were concentrated at the higher end of the continuum. This would suggest that the set of AQ items is less appropriate for measuring degree of autistic traits in the non-ASD group. Furthermore, the result is reasonable given that the AQ was developed to screen adults with Asperger’s syndrome or high-functioning autism, who are more likely to endorse many of the items. During piloting of the AQ, Barron-Cohen (2001a) excluded the items (except items 29 and 30) if non-ASD people selected ‘definitely disagree’ or ‘slightly disagree’ more often than did people with Asperger’s syndrome or high-functioning autism. Consequently, non-ASD respondents would be less likely to endorse items on the AQ and they will thus show worse targeting.

The Rasch analysis supported most of AQ scaling properties but failed to support Barron-Cohen et al.’s (2001a) assumption that AQ measures a single latent variable, namely, the degree of autistic traits. This result is in line with previous research using factor analysis (Austin 2005; Hoekstra et al. 2008; Hurst et al. 2007; Stewart and Austin 2009) and Mokken scaling (Stewart et al. 2015). The hypothesized single latent variable is not consistent with the multidimensional nature of ASD, as expressed in the Diagnostic and Statistical Manual of Mental Disorders, DSM-5 (American Psychiatric Association 2013), or with the fact that Barron-Cohen (2001a) selected the AQ items from the domains in the “triad” of autistic symptoms. The use of a single AQ sum score may therefore not adequately express the multifaceted aspect of ASD.

By reducing the AQ to 12 items from the *Social skill, Attention switching*, and *Communication* domains, we were able to meet both criteria for unidimensionality. Intriguingly, nine of these items (11, 13, 17, 22, 26, 34, 38, 44, and 47) are among the ten items that passed the Mokken scaling test on people with ASD (Stewart et al. 2015). Hoekstra et al. (2008), using CFA, found that the AQ consisted of two second-order factors, one of them including *Social skill, Attention switching* and *Communication*. Using different evaluation methods we thus converged on a similar conclusion: the AQ measures more than one latent variable and consists of an unnecessarily large number of items in order to measure a unidimensional autistic trait. Despite this, a majority of empirical studies use the AQ sum score as the sole measure of an autistic tendency. If the AQ measures a set of (somewhat related) constructs, what exactly does an AQ sum score mean and what consequences does this have for our understanding of autism?

According to the psychometric literature, if the assumption of unidimensionality is violated, any statistical analysis based on it would be misleading. Specifically, estimates of the latent variables and item parameters will generally be biased because of model misspecification, which in turn leads to incorrect decisions on subsequent statistical analysis, such as testing group differences and correlations between latent variables (e.g., Horton et al. 2013).

It should be noted that unidimensionality is a relative matter. The judgment of whether a scale is sufficiently unidimensional should ultimately come from outside the data and be driven by the purpose of measurement, clinical, and theoretical considerations (Andrich 1988; Cano et al. 2011; Rasch 1960).

A pragmatic way to salvage a situation like this would be to treat the AQ sum score as an index, in other words, a formative latent variable (see Simonetto (2012) for an overview). A formative latent variable is defined by a number of non-interchangeable composite indicators, such as income, education, and occupation in the variable socioeconomic status, or weight and height in the variable body mass index. Consequently, a formative latent variable does not exist at a deeper conceptual level than its defining composite indicators (Law et al. 1998). Following this path, AQ sum score will lose content validity and serve as a mere observable outcome and predictor variable.

To what extent, then, can the AQ predict presence of ASD? The person reliability and separation indices of the AQ were adequate, as were the item reliability and separation indices. The AQ has the potential to classify three groups of people (low, average, and high degree of autistic traits) and is at a level of sensitivity required for both group and individual use (Tennant and Conaghan 2007). The AQ may also be able to separate more than ten item difficulty levels, which confirms its item difficulty hierarchy, in other words, its construct validity. The AQ sum score differentiated well between the ASD group and the non-ASD group. The AUC was above that found on similar populations in Britain (e.g. Woodbury-Smith et al. 2005) but lower than that reported in the Netherlands (Wouters and Spek 2011) or Australia (Broadbent et al. 2013). Regarding the AQ domains, the ROC indicated that the domains *Social skill, Attention switching*, and *Communication* had adequate AUC (above 80%), whereas the AUC of *Imagination* was fair and the AUC of *Attention to detail*, though above chance, was poor (below 60%). This is in line with the large proportion (40%) of misfit items in this domain and with previous studies showing that *Attention to detail* is the poorest domain in the AQ for differentiating people with and without ASD diagnoses (Allison et al. 2012; Wouters and Spek 2011).

The AQ logits and sum scores obtained for each individual were highly correlated (r = 0.998); suggesting that summed raw scores adequately reflected true change along the autistic traits continuum that the AQ quantifies. However, it should be borne in mind that the conversion to logits would only be motivated if the sample characteristics are similar to those of the present study. Consequently, Rasch analyses are needed prior to using the AQ on other populations.

### Limitations

Although this study provides an important contribution to our understanding of the AQ and the assessment of autistic traits in people with and without ASD, there are a number of limitations that warrant discussion. First, the groups were not matched for sex and age. The participants in the non-ASD group were younger and included a larger proportion of women than the ASD group. Despite sex and age differences, the DIF analyses showed few discrepancies between the ASD and non-ASD groups. Consistent with previous research, there was no difference between mean AQ sum scores of men and women with ASD (Baron-Cohen et al. 2001a, 2006; Hoekstra et al. 2008).

Moreover, the sample size fulfilled the requirement of stable calibration for Rasch analysis but the subgroups for DIF analysis were too small (see Linacre 2013) to draw a definite conclusion regarding whether, for example, sex- or age-related DIF was present in the items in either the ASD or the non-ASD group. Therefore, any conclusions regarding sex or age differences between groups should be interpreted with caution.

Furthermore, some of the ASD participants attached comments to their questionnaires that it was somewhat challenging for them to complete so many questions. It is reasonable to conclude that some people with ASD, regardless of their motivation to complete the questionnaire, may have lacked the ability to do so. Although all people with ASD registered in the county were invited to participate, the results are only generalizable to those with the ability to complete the AQ questionnaire. This may have less impact on estimated AQ scale properties, because the reported level of autism traits as quantified by AQ is probably an underestimation of the true level in the ASD population. In addition, the non-ASD sample completed the AQ anonymously, which meant that we could not verify whether any of them had an ASD diagnosis or would fall within that category.

## Conclusions

Our findings suggest that several measurement properties of the AQ were good and that it had adequate sensitivity and specificity to distinguish people with ASD from those without ASD, though the AQ sum score did not perform better than the *Social skill* domain alone. Nevertheless, the AQ cannot be described as a unidimensional measurement of the degree to which adults with normal intelligence show autistic traits. Thus, the AQ sum score is probably best regarded as an index. The complementary Rasch analysis showed that the 50-item AQ could be reduced to a 12-item subset with little loss in explanatory power. Following replication on a new sample, this subset of AQ items has the potential to efficiently measure the degree to which adults with and without ASD show autistic traits.

## References

[CR1] Allison C, Auyeung B, Baron-Cohen S (2012). Toward brief “red flags” for autism screening: The short autism spectrum quotient and the short quantitative checklist in 1000 cases and 3000 controls. Journal of the American Academy of Child & Adolescent Psychiatry.

[CR2] American Psychiatric Association (2013). *Diagnostic and statistical manual of mental disorders*,.

[CR3] Andrich D (1988). Rasch models for measurement.

[CR4] Austin EJ (2005). Personality correlates of the broader autism phenotype as assessed by the Autism Spectrum Quotient (AQ). Personality and Individual Differences.

[CR5] Baron-Cohen S, Hoekstra RA, Knickmeyer R, Wheelwright S (2006). The autism-spectrum quotient (AQ)—Adolescent version. Journal of autism and developmental disorders.

[CR6] Baron-Cohen S, Wheelwright S, Hill J, Raste Y, Plumb I (2001). The “Reading the Mind in the Eyes” test revised version: A study with normal adults, and adults with Asperger syndrome or high-functioning autism. Journal of Child Psychology and Psychiatry and Allied Disciplines.

[CR7] Baron-Cohen S, Wheelwright S, Skinner R, Martin J, Clubley E (2001). The autism-spectrum quotient (AQ): Evidence from Asperger syndrome/high-functioning autism, males and females, scientists and mathematicians. Journal of autism and developmental disorders.

[CR8] Bayliss AP, Tipper SP (2005). Gaze and arrow cueing of attention reveals individual differences along the autism spectrum as a function of target context. British Journal of Psychology.

[CR9] Broadbent J, Galic I, Stokes MA (2013). Validation of autism spectrum quotient adult version in an Australian sample. Autism Research and Treatment.

[CR10] Brugha TS, McManus S, Bankart J, Scott F, Purdon S, Smith J, Bebbington J, Jenkins R, Meltzer H (2011). Epidemiology of autism spectrum disorders in adults in the community in England. Archives of General Psychiatry.

[CR11] Cano SJ, Barrett LE, Zajicek JP, Hobart JC (2011). Dimensionality is a relative concept. Multiple Sclerosis.

[CR12] Cohen IL, Gomez TR, Gonzalez MG, Lennon EM, Karmel BZ, Gardner JM (2010). Parent PDD behavior inventory profiles of young children classified according to autism diagnostic observation schedule-generic and autism diagnostic interview-revised criteria. Journal of Autism and Developmental Disorders.

[CR13] Elsabbagh M, Divan G, Koh YJ, Kim YS, Kauchali S, Marcín C (2012). Global prevalence of autism and other pervasive developmental disorders. Autism Research.

[CR01] Engelhard Jr, G. (2013). *Invariant measurement: Using Rasch models in the social, behavioral, and health sciences*. New York, NY: Routledge.

[CR14] Fombonne E (2009). Epidemiology of pervasive developmental disorders. Pediatric Research.

[CR15] Gorsuch RL (1997). Exploratory factor analysis: Its role in item analysis. Journal of Personality Assessment.

[CR16] Hermans EJ, van Wingen G, Bos PA, Putman P, van Honk J (2009). Reduced spontaneous facial mimicry in women with autistic traits. Biological Psychology.

[CR17] Hoekstra RA, Bartels M, Cath DC, Boomsma DI (2008). Factor structure of the broader autism phenotype: A study using the Dutch translation of the autism spectrum quotient (AQ). Journal of Autism and Developmental Disorders.

[CR02] Hoekstra, R. A., Bartels, M., Verweij, C. J., & Boomsma, D. I. (2007). Heritability of autistic traits in the general population. *Archives of Pediatrics & Adolescent Medicine, 161*(4), 372–377. 10.1001/archpedi.161.4.372 17404134

[CR18] Hoekstra RA, Vinkhuyzen AA, Wheelwright S, Bartels M, Boomsma DI, Baron-Cohen S, Posthuma D, van der Sluis S (2011). The construction and validation of an abridged version of the autism-spectrum quotient (AQ-Short). Journal of autism and developmental disorders.

[CR20] Horton M, Marais I, Christensen KB, Bang Christensen Karl, Kreiner Svend, Mesbah Mounir (2013). Dimensionality. Rasch models in health.

[CR21] Hurst R, Mitchell J, Kimbrel N, Kwapil T, Nelson-Gray R (2007). Examinationof the reliability and factor structure of the Autism Spectrum Quotient (AQ) in a non-clinical sample. Personality and Individual Differences.

[CR03] Jackson, S. L., & Dritschel, B. (2016). Modeling the impact of social problem-solving deficits on depressive vulnerability in the broader autism phenotype. *Research in Autism Spectrum Disorders, 21*, 128–138.

[CR22] Karami H (2012). An introduction to differential item functioning. The International Journal of Educational and Psychological Assessment.

[CR23] Keyes KM, Susser E, Cheslack-Postava K, Fountain C, Liu K, Bearman PS (2012). Cohort effects explain the increase in autism diagnosis among children born from 1992 to 2003 in California. International Journal of Epidemiology.

[CR24] Kloosterman PH, Keefer KV, Kelley EA, Summerfeldt LJ, Parker JD (2011). Evaluation of the factor structure of the Autism-Spectrum Quotient. Personality and Individual Differences.

[CR04] Kunihira, Y., Senju, A., Dairoku, H., Wakabayashi, A., & Hasegawa, T. (2006). ‘Autistic’traits in non-autistic Japanese populations: relationships with personality traits and cognitive ability. *Journal of Autism and Developmental Disorders, 36*(4), 553–566. 10.1007/s10803-006-0094-1 16602034

[CR25] Kuo CC, Liang KC, Tseng CC, Gau SSF (2014). Comparison of the cognitive profiles and social adjustment between mathematically and scientifically talented students and students with Asperger’s syndrome. Research in Autism Spectrum Disorders.

[CR26] Kurita H, Koyama T, Osada H (2005). Autism-Spectrum Quotient-Japanese version and its short forms for screening normally intelligent persons with pervasive developmental disorders. Psychiatry and Clinical Neurosciences.

[CR27] Lau WYP, Kelly AB, Peterson CC (2013). Further evidence on the factorial structure of the autism spectrum quotient (AQ) for adults with and without a clinical diagnosis of autism. Journal of Autism and Developmental Disorders.

[CR28] Law KS, Wong CS, Mobley WM (1998). Toward a taxonomy of multidimensional constructs. Academy of Management Review.

[CR29] Linacre JM (1994). Sample size and item calibrations stability. Rasch Measurement Transactions.

[CR30] Linacre JM (2009). A User’s Guide to WINSTEPS.

[CR31] Linacre JM (2013). Differential item functioning DIF sample size nomogram. Rasch Measurement Transactions.

[CR32] Linacre, J. M. (2014). *Winsteps*^*®*^*Rasch measurement computer program*. Beaverton, OR: Winsteps.com.

[CR33] Linacre JM. (2002) Optimizing rating scale category effectiveness. *Journal of Applied Measurement* 3:1 p. 85–106. 11997586

[CR35] Nunnally JC, Bernstein IH (1994). Psychometric theory.

[CR36] Rasch G (1960). Probabilistic models for some intelligence and assessment tests.

[CR37] Rivet TT, Matson JL (2011). Review of gender differences in core symptomatology in autism spectrum disorders. Research in Autism Spectrum Disorders.

[CR38] Robinson EB, Munir K, Munafò MR, Hughes M, McCormick MC, Koenen KC (2011). Stability of autistic traits in the general population: Further evidence for a continuum of impairment. Journal of the American Academy of Child & Adolescent Psychiatry.

[CR39] Rosbrook A, Whittingham K (2010). Autistic traits in the general population: What mediates the link with depressive and anxious symptomatology?. Research in Autism Spectrum Disorders.

[CR40] Simonetto A (2012). Formative and reflective models: State of the art. Electronic Journal of Applied Statistical Analysis.

[CR42] Stewart ME, Allison C, Baron-Cohen S, Watson R (2015). Investigating the structure of the autism-spectrum quotient using Mokken scaling. Psychological Assessment.

[CR43] Stewart ME, Austin EJ (2009). The structure of the Autism-Spectrum Quotient (AQ): Evidence from a student sample in Scotland. Personality and Individual Differences.

[CR44] Stewart ME, Ota M (2008). Lexical effects on speech perception in individuals with “autistic” traits. Cognition.

[CR45] Stewart ME, Watson J, Allcock A-J, Yaqoob T (2009). Autistic traits predict performance on the block design. Autism.

[CR46] Tennant A, Conaghan PG (2007). The Rasch measurement model in rheumatology: What is it and why use it? When should it be applied, and what should one look for in a Rasch paper?. Arthritis Care & Research.

[CR47] Wakabayashi A, Baron-Cohen S, Wheelwright S, Tojo Y (2006). The Autism-Spectrum Quotient (AQ) in Japan: A cross-cultural comparison. Journal of autism and developmental disorders.

[CR48] Woodbury-Smith MR, Robinson J, Wheelwright S, Baron-Cohen S (2005). Screening adults for Asperger Syndrome using the AQ: A preliminary study of its diagnostic validity in clinical practice. Journal of Autism and Developmental Disorders.

[CR49] World Health Organization (1993). International classification of disease and related health problems 10th Revision.

[CR50] Wouters SG, Spek AA (2011). The use of the Autism-spectrum Quotient in differentiating high-functioning adults with autism, adults with schizophrenia and a neurotypical adult control group. Research in Autism Spectrum Disorders.

[CR51] Wright BD (1993). Logits?. Rasch Measurement Transactions.

[CR52] Youden WJ (1950). Index for rating diagnostic tests. Cancer.

